# The Coexistence of *Klebsiella pneumoniae* and *Candida albicans* Enhanced Biofilm Thickness but Induced Less Severe Neutrophil Responses and Less Inflammation in Pneumonia Mice Than *K. pneumoniae* Alone

**DOI:** 10.3390/ijms252212157

**Published:** 2024-11-12

**Authors:** Pornpimol Phuengmaung, Chiratchaya Chongrak, Wilasinee Saisorn, Jiradej Makjaroen, Uthaibhorn Singkham-in, Asada Leelahavanichkul

**Affiliations:** 1 Department of Microbiology, Faculty of Medicine, Chulalongkorn University, Bangkok 10330, Thailand; pphuengmaung@gmail.com (P.P.); chiratchayachr@gmail.com (C.C.); wsaisorn@gmail.com (W.S.); 2 Center of Excellence on Translational Research in Inflammation and Immunology (CETRII), Department of Microbiology, Faculty of Medicine, Chulalongkorn University, Bangkok 10330, Thailand; 3 Medical Microbiology, Interdisciplinary and International Program, Graduate School, Chulalongkorn University, Bangkok 10330, Thailand; 4 Department of Transfusion Medicine and Clinical Microbiology, Faculty of Allied Health Sciences, Chulalongkorn University, Bangkok 10330, Thailand; jiradejmak@gmail.com; 5 Faculty of Medical Technology, Rangsit University, Pathum Thani 12000, Thailand; 6 Division of Nephrology, Department of Medicine, Faculty of Medicine, Chulalongkorn University, Bangkok 10330, Thailand

**Keywords:** Interkingdom biofilms, *Klebsiella pneumoniae*, *Candida albicans*, pneumonia model

## Abstract

Due to the possible coexistence of *Klebsiella pneumoniae* (KP) and *Candida albicans* (CA), strains of KP and CA with biofilm production properties clinically isolated from patients were tested. The production of biofilms from the combined organisms (KP+CA) was higher than the biofilms from each organism alone, as indicated by crystal violet and z-stack immunofluorescence. In parallel, the bacterial abundance in KP + CA was similar to KP, but the fungal abundance was higher than CA (culture method), implying that CA grows better in the presence of KP. Proteomic analysis was performed to compare KP + CA biofilm to KP biofilm alone. With isolated mouse neutrophils (thioglycolate induction), KP + CA biofilms induced less prominent responses than KP biofilms, as determined by (i) neutrophilic supernatant cytokines (ELISA) and (ii) neutrophil extracellular traps (NETs), using immunofluorescent images (neutrophil elastase, myeloperoxidase, and citrullinated histone 3), *peptidyl arginine deiminase 4* (*PAD4*) expression, and cell-free DNA. Likewise, intratracheal KP + CA in C57BL/6 mice induces less severe pneumonia than KP alone, as indicated by organ injury (serum creatinine and alanine transaminase) (colorimetric assays), cytokines (ELISA), bronchoalveolar lavage fluid parameters (bacterial culture and neutrophil abundances using a hemocytometer), histology score (H&E stains), and NETs (immunofluorescence on the lung tissue). In conclusion, the biofilm biomass of KP + CA was mostly produced from CA with less potent neutrophil activation and less severe pneumonia than KP alone. Hence, fungi in the respiratory tract might benefit the host in some situations, despite the well-known adverse effects of fungi.

## 1. Introduction

Biofilms are well known as a structured community of microorganisms covered with extracellular polymeric substances (EPSs) containing polysaccharides, proteins, and DNA [1]. The rising ability of biofilm formation through several functional genes increases virulence and induces drug resistance. *Klebsiella pneumoniae* (KP), a Gram-negative bacterium in the Enterobacteriaceae family, may colonize several parts of the human body, including the nasopharynx, intestinal mucosa, and skin as the normal microbiota [2]. Indeed, KP causes a wide range of infections, including bloodstream infection, respiratory system infection, and urinary tract infection [2]. Bacteria in sessility (attached bacteria in one place that are usually non-motile), referred to as the sessile form, in biofilms are more resistant to antibiotics and host immunity than planktonic cells (free-floating bacteria), partly through the higher production of fimbriae, polysaccharides, and efflux pumps [3,4]. Different from isolated living bacteria in planktonic form, a group of sessile bacteria regulates the expression of several genes in response to the alteration of cell population density, also known as the quorum sensing (QS) system, which is associated with biofilm formation [5]. Biofilm production is a major virulence determinant, and bacteria with high levels of biofilm production are frequently described together with multidrug-resistant properties [6]. Infections with biofilm-producing bacteria that are attached to living and nonliving surfaces, including non-device-related biofilms (cystic fibrosis, infective endocarditis, infectious nephrolithiasis, necrotizing fasciitis, osteomyelitis, tonsillitis, periodontitis, and dental plaque) and device-related infections (indwelling medical devices), are usually more difficult to treat than common bacteria [7].

Interestingly, biofilm-related infection may be caused by a single microbe or mixed microorganisms, and the mixed organisms can be a combination of different species within the same phylum (multi-species biofilms) [8] or different phyla (interkingdom biofilms) [9]. Approximately 40–80% of bacteria in natural settings exist as biofilms, most of which are mixed-species biofilms due to the multiple organisms in the microenvironment [10]. For biofilm formation, the lipopolysaccharides (LPS) and cell wall glyco-polymers of Gram-negative and Gram-positive bacteria, respectively, are similarly important for the initial adhesion of the cells to the surfaces [11]. After that, microcolony formation is assisted by different extracellular matrices and molecules depending on the microorganisms, for example, Psl polysaccharide (*Pseudomonas aeruginosa*), vibrio polysaccharide (*Vibrio cholera*), capsular polysaccharides with fimbriae (KP), exopolysaccharide (*Basillus subtilis*), polysaccharide intercellular adhesion with extracellular DNA or cell wall-anchored proteins (*Staphylococcus aureus*), and aggregated substances with enterococcal surface proteins and pili (*Enterococcus* spp.) [11]. Motility inhibition through downregulation of fimbriae or pilli and elevation of c-di-GMP (Bis-3′-5′–cyclic dimeric guanosine monophosphate, an important secondary messenger regulating many bacterial motility and activity) is also important for biofilm formation in both Gram-positive and Gram-negative bacteria [12]. Despite extensive studies in bacterial biofilms, data on fungal biofilms and mixed bacterial–fungal biofilms are still limited. Interkingdom biofilms may exist only between some pairs of the organisms because of the competition for food resources in nature [13]. As such, combined bacterial–fungal biofilms are commonly found in the lung, urinary tract, wound, and medical devices, partly because some fungi, especially *Candida albicans* (CA), are part of the normal microbiota in humans and can coexist with bacteria in various parts of the body [14]. However, the coexistence of *C. albicans* with various bacteria, such as *Pseudomonas aeruginosa*, *Staphylococcus aureus*, and *Escherichia coli*, can lead to the development of interkingdom biofilms. These interactions can be synergistic or antagonistic, depending on internal and external factors [15].

Due to the limited data on mixed *Klebsiella–Candida* (KP + CA) biofilms and the high prevalence of *Klebsiella* pneumonia in patients [16], biofilms from KP and CA were selected for this study. Capsular polysaccharides and fimbriae are significant virulence factors that are responsible for adhesion and contribute to biofilm formation of *K. pneumoniae*, whereas biofilms from other bacteria are wrapped with extracellular polymeric substances (EPS) that are secreted by themselves to specifically produce biofilms [17]. The overexpression of type 1 fimbriae (fim gene cluster) affects host immune evasion, whereas type 3 fimbriae (mrk gene cluster) lead to biofilm formation on the abiotic surface [17]. In addition, hyperproduction of polysaccharide capsules (cps gene cluster) induces biofilm adhesion and biofilm maturation [18]. For *Candida* biofilms, the hyphae form of fungi is important for biofilm architectural stability, which is regulated by several quorum-sensing molecules (tyrosol and farnesol) [19,20,21]. A previous study on KP + CA biofilms demonstrates some proteins from KP that decrease biofilm biomass [22], while trehalose from bacteria and fungi [23] possibly enhances biofilm maturation [24]. Therefore, understanding the molecular mechanisms of interkingdom biofilm formation may be crucial for novel treatment strategies for biofilm-associated infections. For example, the biofilm-producing synergy of *P. aeruginosa* plus *C. albicans* is possibly due to enhanced *Pseudomonas* biofilm production from some fungal molecules [25], and the blockage of these molecules may be beneficial.

Based on *Pseudomonas–Candida* synergy on biofilm production [25], we hypothesized that KP + CA biofilms might also demonstrate an additive effect. Then, strains of KP and CA with previously known biofilm production properties clinically isolated from patients [25,26,27] were selected to be tested as a proof-of-concept study. Additionally, proteomic analyses were performed to explore the differences between (i) KP in sessile form (biofilms) and planktonic form (free organisms), and (ii) KP + CA biofilms and KP biofilms. Moreover, neutrophil responses and a murine pneumonia model were also conducted to test the differences between KP + CA and KP or CA alone.

## 2. Results

### 2.1. Candida Elevated Thickness of the Interkingdom Biofilms

**Figure 1:**
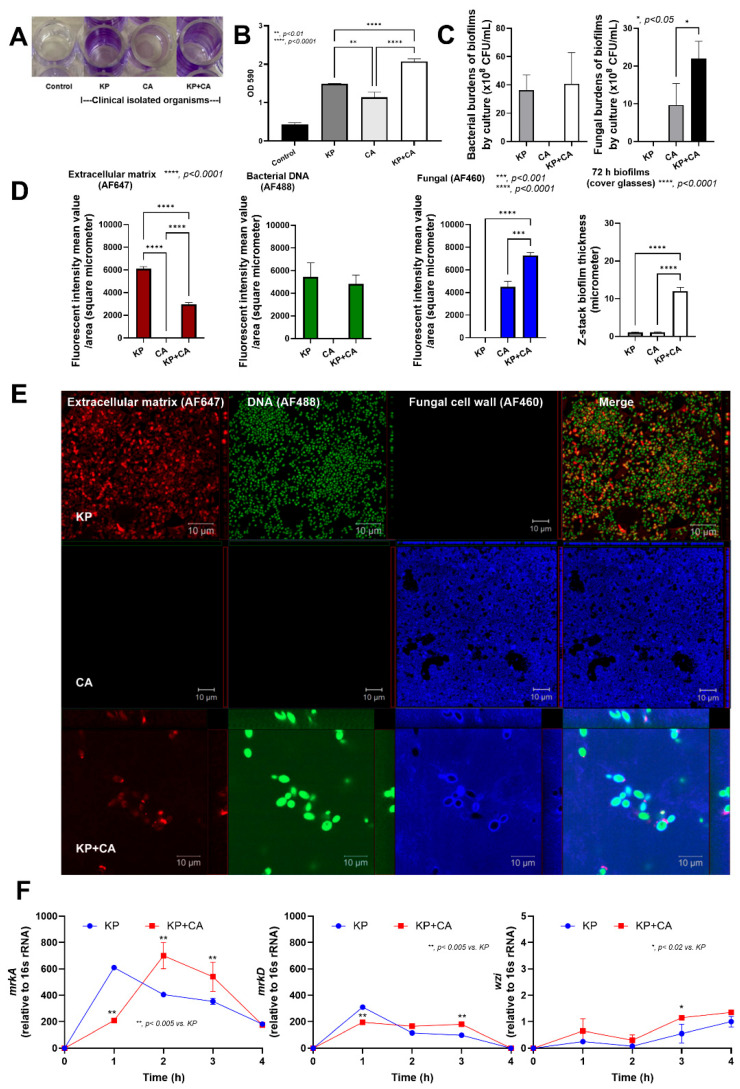
Crystal violet-stained biofilms in 96-well-plates with representative pictures at 72 h post-incubation of the control group (no organisms), *Klebsiella pneumoniae* (KP), *Candida albicans* (CA), and combined organisms (KP + CA) (**A**,**B**) are demonstrated. Characteristics of the biofilms from KP, CA, and KP + CA as indicated by microbial burdens (culture methods) (**C**), intensity score from the fluorescent-stained cover glasses to detect bacterial extracellular matrix (ECM) using AF647 (red color fluorescence), nucleic acid stained by SYTO9 (AF488; green color fluorescence), and fungal cell wall using calcofluor white (AF460; blue color fluorescence against chitin on fungal cell wall) with representative fluorescent images (**D**,**E**) are also demonstrated. Expression of some bacterial genes from biofilms of KP alone and KP + CA in the early phase of biofilm production (within 4 h post-incubation) (**F**) was shown. Data were retrieved from triplicate independent experiments. Fluorescent intensity was analyzed by the ZEN imaging software (Carl Zeiss, Oberkochen, Germany).

Clinically isolated strains of *K. pneumoniae* (KP) and *C. albicans* (CA) from patients in the King Chulalongkorn Memorial Hospital selected from our previous publications [25,26,27] were used. With crystal violet staining at 72 h post-incubation, both KP and CA produced biofilms when compared with the control (media without organisms) (Figure 1A,B). The biofilms from mixed organisms (KP + CA) were more prominent than those from KP alone, as indicated by crystal violet staining (Figure 1A,B). With the culture method, the bacterial abundance in KP + CA was similar to KP alone, while the fungal abundance of KP + CA was higher than that of CA alone (Figure 1C). Because the bacterial abundance of KP + CA was approximately twofold higher than the fungal abundance (Figure 1C), there was no inhibitory effect on bacterial growth from fungi. Despite more prominent biofilms in KP + CA with crystal violet staining (Figure 1A,B), confocal electron microscopy demonstrated a lower extracellular matrix (ECM) in KP + CA compared with KP (Figure 1D,E), supporting better growth of fungi in the present of some bacteria [28]. There were no immunofluorescent signals of ECM in CA alone (Figure 1D,E), although faint crystal violet staining was observed (Figure 1A,B), indicating slight biofilm production from CA and highlighting the limitations of crystal violet staining in biofilm detection [29]. Clear budding yeast cells, positive for both DNA and fungal cell wall markers, were observed in KP + CA but not in CA alone, while there were positive cell wall markers with negative DNA staining in CA alone (Figure 1D,E). It is possible that intact cell walls in CA interfere with an influx of DNA-staining dye [25,26] (negative DNA staining), while budding-induced cell wall defects [30] in KP + CA allow color influx into the fungal cells (positive DNA staining). Interestingly, the biofilm thickness (z-stack imaging) in KP + CA was more prominent than in KP or CA alone (Figure 1D,E). After that, gene expression analysis was performed within 4 h post-incubation (an early phase of biofilm production). As such, both KP + CA and KP similarly upregulated fimbriae type 3 genes (*mrkA* and *mrkD*) and capsule-related genes (*wzi*) at 1–3 h and 3–4 h post-incubation, respectively (Figure 1F), despite a lower extracellular matrix in KP + CA compared with KP (Figure 1D,E). Notably, the greater thickness of KP + CA biofilms compared to KP alone, along with the lower ECM content in KP + CA than in KP (Figure 1D,E), suggested that CA contributes to KP + CA biofilm thickness. There was no expression of these genes at 6, 12, and 24 h post-incubation, implying rapid RNA alteration at an early phase of biofilm formation.

**Figure 2:**
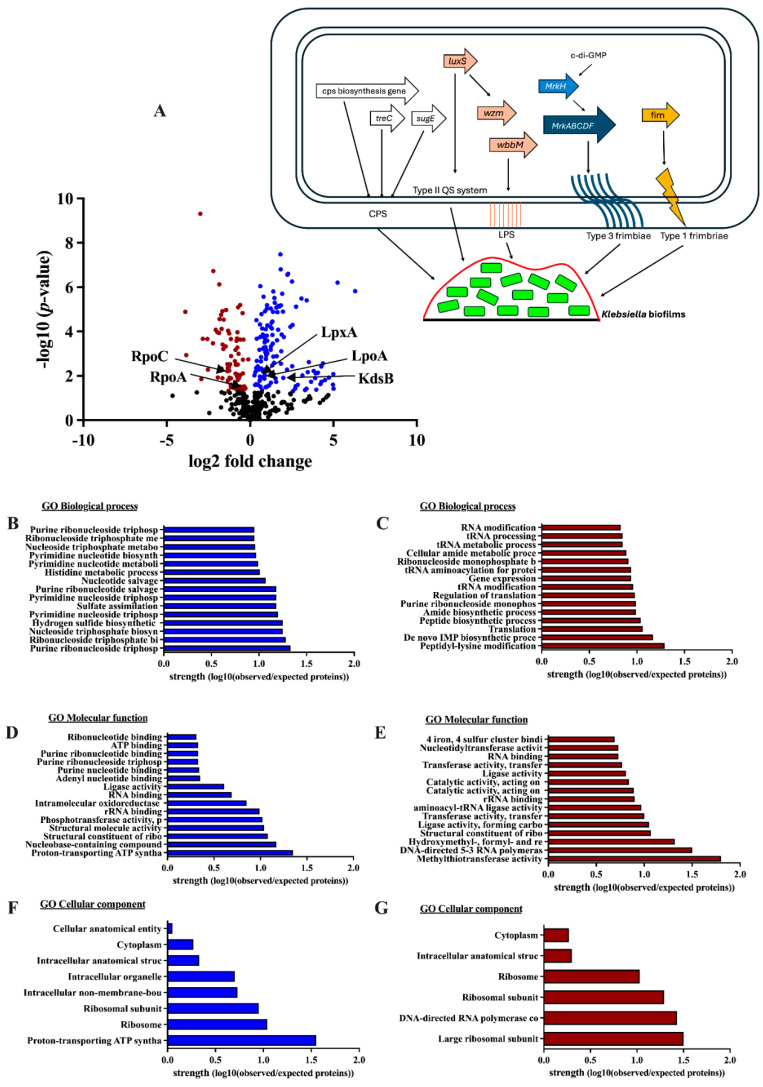
The proteomic analysis of the biofilms from *Klebsiella pneumoniae* (KP, consisting of sessile KP with extracellular matrix) relative to the proteins from KP in planktonic form (non-biofilm-producing form without extracellular matrix), as indicated by a volcano plot with a picture indicating the functions of the important proteins for KP biofilm synthesis (**A**), and the biological analysis, using DAVID v2021q4 Bioinformatics resource 2021 software for protein gene ontology (GO), of the upregulated proteins (blue color) and downregulated proteins (brown color) (**B**–**G**), is demonstrated. cps, capsular polysaccharide; treC, trehalose-6-phosphate hydrolase; sugE, intima protein; LuxS, S-ribosylhomocysteine lyase; wzm, transport polymerase protein; wbbm, glycosyl transferase; MrkH, mannose-resistant *Klebsiella*-like H (a c-di-GMP-dependent transcriptional activator; a biofilm switch molecule); MrkABCDF, mannose-resistant *Klebsiella*-like ABDCF; fim, fimbriae; LPS, lipopolysaccharide; LpxA, UDP-N-acetylglucosamine acyltransferase; LpoA, penicillin-binding protein activator; KdsB, 3-deoxy-manno-octulosonate cytidyltransferase; RpoA, DNA-directed RNA polymerase subunit alpha; RpoC, DNA-directed RNA polymerase subunit beta. In the hypothetic picture, white color arrows indicate capsular polysaccharide (CPS)-associated proteins [18], orange arrows are proteins of the type II quorum sensing (QS) system and LPS synthesis [31], light and dark blue arrows are type III fimbriae-associated proteins (fimbriae-associated biofilms), and the bright orange arrow is type I fimbriae (non-biofilm fimbriae) [17].

**Figure 3:**
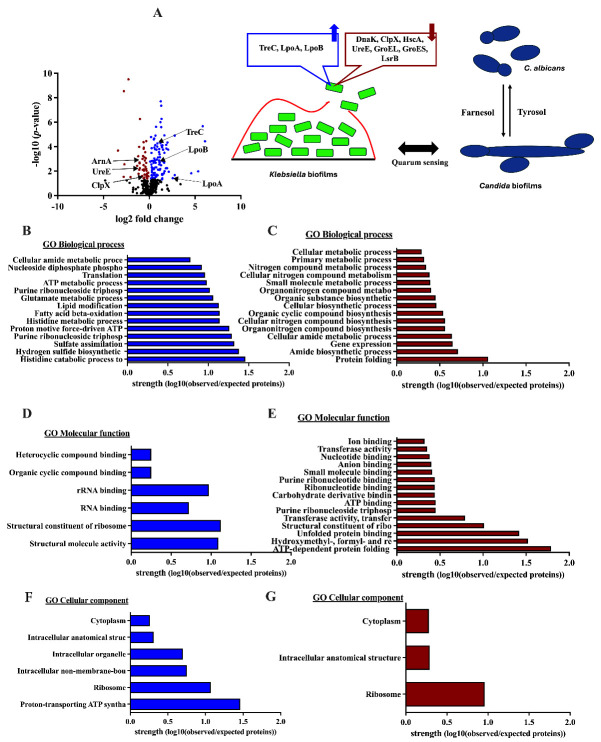
The proteomic analysis from biofilms of the mixed *Klebsiella pneumoniae* plus *Candida albicans* (KP + CA) relative to KP alone, as indicated by a volcano plot with a picture indicating the functions of the important up- and downregulated proteins with a hypothetic picture of the possible connection (quorum sensing) between the Candida quorum sensing molecules, including farnesol (inhibitor of fungal filamentation) and tyrosol (activator of fungal filamentation) (**A**), with the biological analysis, using v2021q4 DAVID Bioinformatics resource 2021 software for protein gene ontology (GO), of the upregulated proteins (blue color) and downregulated proteins (brown color) (**B**–**G**), is demonstrated. cps, capsular polysaccharide; treC, trehalose-6-phosphate hydrolase; sugE, intima protein; LuxS, S-ribosylhomocysteine lyase; wzm, transport polymerase protein; wbbm, glycosyl transferase; fim, fimbriae; LPS, lipopolysaccharide; LpoA, penicillin-binding protein activator LpoA; LpoB, penicillin-binding protein activator LpoB; ArnA, bifunctional polymyxin resistance protein ArnA; UreE, urease accessory protein UreE; ClpX, ATP-dependent Clp protease ATP-binding subunit ClpX; DnaK, chaperone protein DnaK; HscA, chaperone protein HscA; GroES, chaperonin GroEL; LsrB, autoinducer 2-binding protein LsrB.

Then, proteomic analysis was performed in two sets, including (i) proteins of the KP biofilms (sessile-form bacteria with extracellular matrix proteins) in relation to KP in the planktonic form (non-biofilm-producing bacterial form) (Figure 2), and (ii) proteins of KP + CA biofilms in relation to KP biofilms at 72 h post-incubation (Figure 3), to explore the possible differences between groups. As such, the volcano plot of proteins from the KP biofilms compared with planktonic-form KP demonstrated that 135 upregulated proteins and 77 downregulated proteins are correlated with biofilm formation (Figure 2A). Among the 135 upregulated proteins, KdsB, LpxA, and LpoA are proteins associated with LPS synthesis, which may be associated with an early phase of biofilm attachment [31]. In addition, the upregulation of chaperones (GroES, GupE, HtpG, and Tig), copper homeostasis (CutC), iron (Fe) homeostasis (CyaY), and sulfur metabolism (CysH, CysI, and CysJ) may be used by sessile KP for cell homeostasis [32,33,34]. In contrast, most of the downregulated proteins were those involved in tRNA modification (TrmA, Tgt, TruB, YleA, YfgB, YliG, and SirA), peptide biosynthesis (such as RpmD, RpmF, RpmG, RpmH, RplB, RplO, RpsI, RpsK, RpsL, RpsR, and RpsU), and RNA metabolic processes (such as RpoA, RpoB, and RpoC), suggesting a reduction in cellular metabolism in sessile KP compared to free-form planktonic KP [35,36]. Decreased autoinducer 2 (AI-2)-binding proteins (LsrB and LsrG) indicated reduced planktonic-form KP during biofilm formation [37]. To understand the biological deregulation of aberrantly expressed proteins, the differentially quantified proteins were used and further analyzed through functional annotation with DAVID v2021q4 Bioinformatics resource 2021 software for protein gene ontology (GO) and pathway analysis. The significantly upregulated proteins (blue color) and downregulated proteins (brown color) in sessile KP biofilms compared to planktonic KP are illustrated in Figure 2B–G. In short, downregulated cell metabolisms and increased synthesis of the protein components of biofilms were demonstrated by proteomic analysis of sessile KP compared with planktonic KP.

For proteins from KP + CA biofilms compared with KP biofilms, the volcano plot demonstrated 413 proteins in KP + CA biofilms with approximately 100 biofilm-formation-associated proteins, including an increase in (i) modulators of capsular polysaccharide production (an enzyme that divides trehalose-6-phosphate into glucose and glucose-6-phosphate; TreC) [38], (ii) penicillin-binding protein activators (LpoA and LpoB) [39], and (iii) the proteins of the proton-transporting ATP synthase complex (AtpC, AtpG, and AtpA) [35], which may interact with the fungi (Figure 3A,B). In contrast, the proteins in lipid A modification (ArnA) and protein folding (DnaK, ClpX, HscA, UreE, GroEL, and GroES) [35] were downregulated (Figure 3A). With the quantified protein GO pathway, downregulated proteins in KP + CA biofilms compared with KP biofilms were associated with protein folding, gene expression, and cellular metabolic processes, whereas upregulated proteins were correlated with structural molecules, ATP synthase complexes, and amino acid metabolism (Figure 3B–G). Hence, reduced ECM without bacterial DNA depletion (fluorescent staining) and altered microbial abundance (the culture method) in KP + CA compared with CA alone (Figure 1C–E) may be due to the downregulation of protein folding and cell metabolic processes (Figure 3B–G). More mechanistic studies on this topic would be interesting.

### 2.2. Less Prominent Neutrophil Responses Against K. pneumoniae Plus C. albicans (KP + CA) Biofilms Compared to Those from KP Alone (the In Vitro Test)

**Figure 4:**
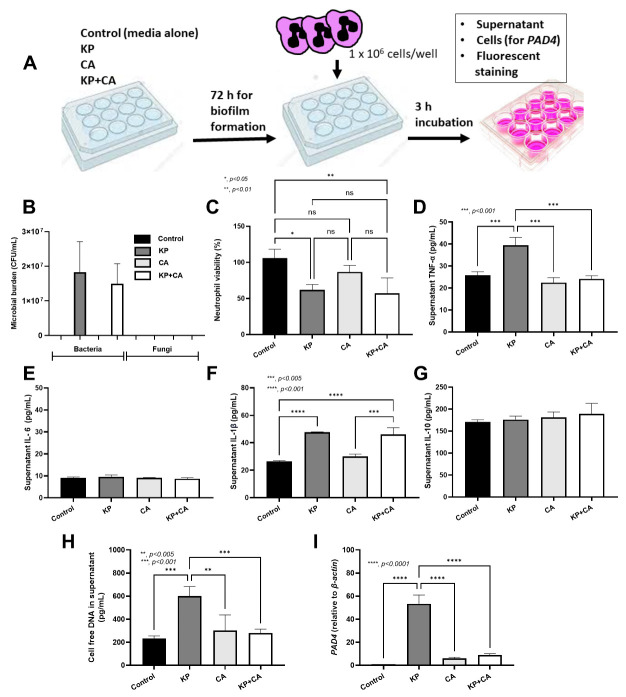
Schema of the experiments creating biofilms of *K. pneumoniae* (KP), *C. albicans* (CA), combined organisms (KP + CA), or control (culture media without organisms) (**A**) and the characteristics of the experiments as indicated by microbial burdens (culture for bacteria and fungi) (**B**), neutrophil viability (**C**), supernatant cytokines (TNF-α, IL-6, IL-1β, and IL-10) (**D**–**G**), cell-free DNA (**H**), and *peptidyl arginine deiminase 4* (*PAD4*) expression (**I**) are demonstrated. Data were retrieved from triplicate independent experiments.

**Figure 5:**
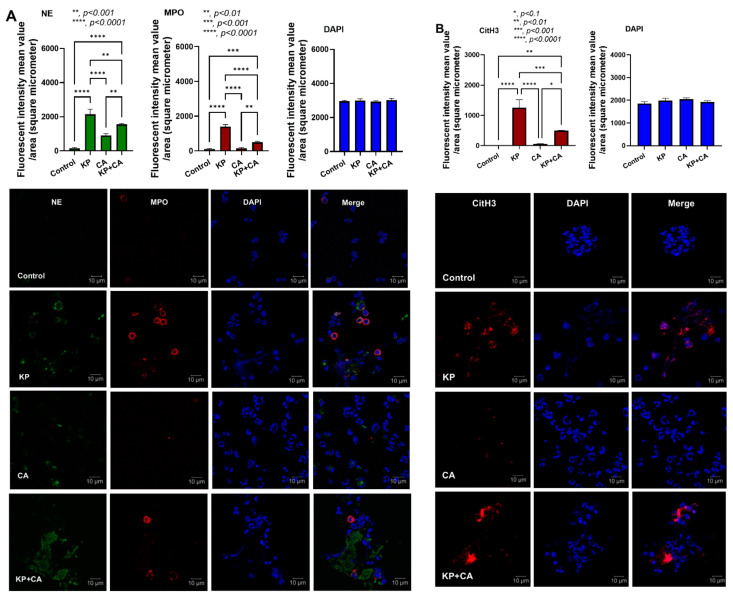
The neutrophil extracellular traps (NETs) in the biofilms of *K. pneumoniae* (KP), *C. albicans* (CA), combined organisms (KP + CA), or control (culture media without organisms) as indicated by the immunofluorescence of neutrophil elastase (NE; green color) and myeloperoxidase (MPO; red color) with the representative immunofluorescent pictures (**A**) and the immunofluorescence for citrullinated histone 3 (CitH3) with the representative pictures (**B**) are demonstrated. Notably, 4′,6-diamidino-2-phenylindole (DAPI; blue color) was used for the nuclear staining. Data were retrieved from triplicate independent experiments. ***, *p* ≤ 0.001.

ECM production in KP + CA biofilms was higher than in CA biofilms (Figure 1C–E). The more prominent thickness of KP + CA biofilms was higher than KP biofilms alone (Figure 1D, far right), which may protect KP + CA from immune cells better than KP alone [40]. Then, neutrophils were incubated with biofilms for 3 h before the determination of several parameters (Figure 4A). As such, there was no significant difference in bacterial burden between KP and KP + CA biofilms (Figure 4B), supporting the biofilm protective effect against immune cells [41]. In parallel, there was negative fungal culture from both CA and KP + CA biofilms (Figure 4B), despite the detectable fungi from the biofilms without neutrophils in the in vitro system (Figure 1C), implying some level of neutrophil fungicidal activity [42]. The neutrophil viability was reduced only in KP and KP + CA but not CA alone (Figure 4C), supporting possible higher neutrophil stresses against bacteria than fungi [43]. None of the neutrophil parameters in CA alone were different from the controls (Figure 4C–I). In comparison with KP alone, KP + CA biofilms induced lower supernatant TNF-α, but not other cytokines (IL1β, IL-6, and IL-10), together with less prominent neutrophil extracellular traps (NETs), as determined by cell-free DNA, *PAD4* expression, and immunofluorescent staining for neutrophil elastase (NE), myeloperoxidase (MPO), and citrullinated histone 3 (CitH3) (Figure 4H,I and Figure 5A,B). Hence, the increased thickness of KP + CA biofilms compared to KP biofilms may be associated with more effective neutrophil inhibition. Further explorations of this topic hold significant interest.

### 2.3. Intratracheal Administration by K. pneumoniae Plus C. albicans (KP + CA) Demonstrated Less Severe Pneumonia Than Kp Alone in Mice

**Figure 6:**
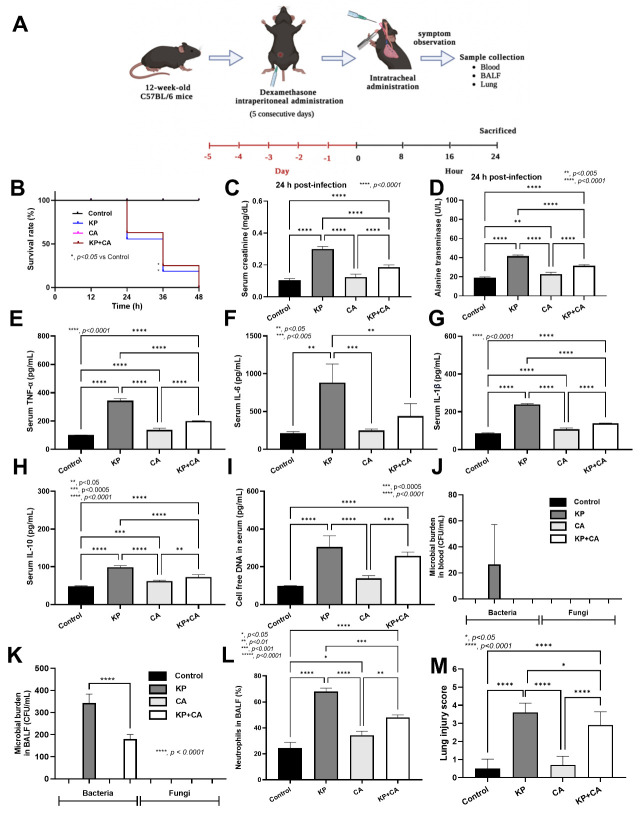
Schema of the experiments of the pneumonia model in C57BL/6 mice with dexamethasone administration for 5 days before intratracheal administration with *K. pneumoniae* (KP), *C. albicans* (CA), combined organisms (KP + CA), or control (vehicle without organisms) as indicated by survival analysis (**A**), serum creatinine (**B**), serum alanine transaminase (**C**), serum cytokines (TNF-α, IL-6, IL-1β, and IL-10) (**D**–**H**), cell-free DNA (**I**), microbial burdens in blood and in bronchoalveolar lavage (BALF) (**J**,**K**), neutrophils in the BALF (**L**), and the lung injury score based on hematoxylin- and eosin-stained histopathology (**M**) are demonstrated. (n = 10/group and n = 6–8/group for others.). *, *p* ≤ 0.05.

**Figure 7:**
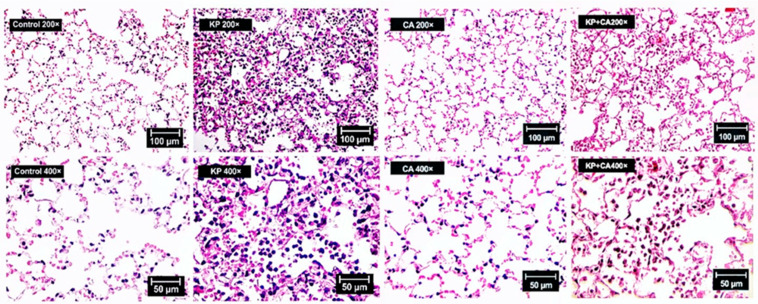
Representative pictures of hematoxylin- and eosin-stained histopathology of mouse lungs with intratracheal administration by *K. pneumoniae* (KP), *C. albicans* (CA), combined organisms (KP + CA), or control (no organisms) are demonstrated to indicate lung consolidation (white blood cell infiltration in the alveolar space) in KP-only and KP + CA mice.

**Figure 8:**
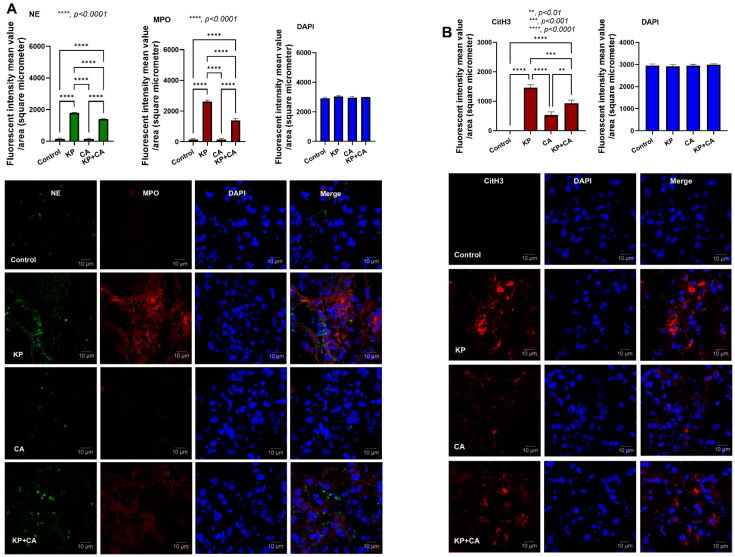
Neutrophil extracellular traps (NETs) in the lung lesion of pneumonia mice with intratracheal administration by *K. pneumoniae* (KP), *C. albicans* (CA), combined organisms (KP + CA), or control (no organisms) as indicated by the immunofluorescence of neutrophil elastase (NE; green color), myeloperoxidase (MPO; red color), and citrullinated histone 3 (CitH3; red color) with representative pictures (**A**,**B**) are demonstrated. Notably, DAPI (blue color) was used for nuclear staining (n = 6–8/group).

The natural presence of *Candida* spp. in the human respiratory tract, partly from fungal translocation from the gastrointestinal system [44], may induce bacteria–fungi biofilms, which might be more difficult to treat than bacterial infection alone [25]. Here, dexamethasone induction in mice before intratracheal administration with KP alone or KP + CA demonstrated similar mortality and organ injuries (serum creatinine and alanine transaminase) (Figure 6A,B). Meanwhile, CA alone did not induce any injuries in mice (Figure 6B–M, Figure 7 and Figure 8A,B), but slightly increased the number of neutrophils in the bronchoalveolar lavage fluid (BALF) (Figure 6L). In comparison with KP alone, KP + CA induced less severe injury, as indicated by the serum cytokines, BALF parameters (microbial burdens and neutrophil abundance), lung injury score, and NETs in the lung tissue (using the immunofluorescence of NE, MPO, and CitH3) (Figure 6B–M, Figure 7 and Figure 8A,B).

## 3. Discussion

Although *K. pneumoniae* (KP) and *C. albicans* (CA) are common pathogens, research on the interaction between these organisms in pneumonia is still limited. Most studies have focused on KP as a single pathogen or combined KP with other bacteria or viruses in pneumonia models [45,46,47,48,49]. Because CA is commonly found in the respiratory system, partly due to translocation from the gastrointestinal tract [50], the copresence of KP with CA is clinically possible. As such, bacterial–fungal biofilms may enhance resistance against host immunity and antimicrobial agents. These interkingdom biofilms may protect bacteria (aerobes and anaerobes) from immunity, as demonstrated in the skin, lung, and gastrointestinal systems [17,51]. In KP biofilms, *mrkA* and *mrkD*, forming the major subunit of type 3 fimbriae used for abiotic surface attachment during rapid biofilm formation [3], were rapidly upregulated as early as 1 h after incubation before declining, as measured by PCR, while MrkA and MrkD in the protein levels were still identified through proteomic analysis at 72 h post-incubation. These data support the possible different time points between RNA expression and protein synthesis [52]. The early expression of *mrkA* and *mrkD* indicates possible rapid biofilm formation, which may occur too early to detect the biofilm proteins using standard methods like crystal violet and immunofluorescence, which need 48–72 h after incubation (Figure 1A–E). In bacterial–fungal biofilms, bacterial ECM may stimulate fungal farnesol, preventing the transformation from mold into yeast and helping bacterial attachment to the surface of hyphae [53,54]. Here, KP enhanced CA proliferation (culture method in Figure 1C), while KP biofilm production was reduced with the presence of CA (immunofluorescence in Figure 1D,E). These results implied an interaction between KP and CA. Indeed, CA produces farnesol and tyrosol to inhibit and stimulate filamentation, respectively [20,55]. Other fungal molecules may interfere with KP biofilm production (hypothetic picture on Figure 3A). Then, proteomic analysis was performed. Accordingly, KP + CA biofilms more prominently upregulated LpoA and LpoB (cell wall synthesis) in proteomic analysis [39] than KP biofilms, whereas trehalose (TreC) only presented in KP + CA. Indeed, trehalose (a nonreducing sugar containing two glucose subunits) can be produced by both bacteria and fungi for carbon sources [56,57]. In mature CA biofilms, trehalose leads to biofilm dispersal and microbial spreading with sepsis [22,26]. Further studies on the enhanced trehalose production from combined KP + CA and the function of trehalose on interkingdom biofilms would be interesting. However, the interaction between bacteria and fungi might be very dynamic, depending on the infected sites (different reactions in the different organs from various microenvironments) and time (differences between early- and late-phase biofilms).

Despite this possible complexity, our model demonstrated differences between KP + CA and KP alone in at least three parts of the experiments. First, KP + CA biofilms were thicker than KP biofilms, as shown by immunofluorescence. Second, there were less prominent neutrophil responses in KP + CA than in KP alone, despite similar bacterial abundance (culture method in Figure 1C), possibly due to more prominent biofilm thickness in KP + CA biofilms. Indeed, neutrophils, the most abundant immune cells in human circulation [58,59], reduce biofilm-associated bacterial dissemination [60] and effectively eradicate small microorganisms [61,62,63,64]. Third, KP + CA induced less severe pneumonia than KP alone. Although pneumonia mice rapidly died within 2 days, the upregulation of biofilm-associated genes was demonstrated at 1–4 h after incubation. Notably, sessile bacteria in biofilms induced less prominent immune responses than the planktonic form [17,65]. Despite the thicker biofilms in the KP + CA group (the in vitro test without neutrophils), KP + CA induced less severe immune responses (the in vitro test with neutrophils) and pneumonia (in mice). Then, predicting the clinical impacts of mixed organism biofilms may require in vitro testing with immune cells or animal studies, rather than relying on in vitro biofilm determination alone. Interestingly, the effective immune responses (complement, acute phase proteins, and chemotaxis) in mice induced several discordances between the in vitro neutrophil responses and the pneumonia model. For example, the bacterial burdens and cell-free DNA in the serum of KP + CA mice were significantly lower than KP alone, while these levels between groups in vitro were not different. Additionally, the neutrophil abundance in the BALF of KP + CA was lower than that of KP alone, while the neutrophil viability in vitro between groups was similar. The in vivo test may be the most suitable tool for determining the impacts of mixed organism biofilms; however, the in vitro test with immune cells may be easier to perform for determining the virulence direction of mixed biofilms. The development of in vitro models that better resemble in vivo responses is needed. Because only one strain of KP and CA with biofilm production properties was tested here as a proof-of-concept study, the combination between KP and CA with other properties and other pairs of organisms is also interesting. Notably, fungi are not normal microbiota in the lung but can easily be transferred from the gastrointestinal tract, preferring to stay in the respiratory tract in some people, especially with chronic respiratory diseases (asthma, chronic obstructive pulmonary disease, and cystic fibrosis) [66]. Due to different fungal abundances in the respiratory system of an individual person, fungal abundance determination may be useful as a predictor in some conditions. Moreover, additional knowledge on the pathophysiology of mixed bacterial–fungal infections may be useful for novel treatment strategies. Although fungi may affect several diseases (pneumonia, Helicobacter pylori [51], and asthma [50]) and fungi themselves induce several complications [67], the discovery of fungal benefits, at least in some situations [68], could pave the way for novel treatments. More studies are warranted.

## 4. Materials and Methods

### 4.1. Bacterial and Fungal Isolates and Biofilm Characterization

The sample accession process of clinically isolated *Klebsiella pneumoniae* (KP) and *Candida albicans* (CA) from patients was approved by the ethical institutional review board, Faculty of Medicine, Chulalongkorn University (approval protocol No. 001/2022) according to the Declaration of Helsinki with written informed consent. Then, KP and CA were grown on Tryptic soy agar (TSA; DifcoTM, Becton, NJ, USA) and Sabouraud dextrose agar (SDA; Oxoid Ltd., Basingstoke, Hampshire, UK), respectively, at 37 °C overnight to provide enough organisms for further procedures. Then, KP or CA was cultured in Tryptic soy broth (TSB; DifcoTM, Franklin Lakes, NJ, USA) or Sabouraud dextrose broth (SDB; Oxoid, Hampshire, UK), respectively, for 24 h at 37 °C overnight before adjusting to a turbidity of 0.5 McFarland standard (approximately 10^8^ CFU/mL) and further incubated for 72 h to grow the single-organism biofilms (KP and CA biofilms). For mixed-organism biofilms (KP + CA), half of the volume of the 0.5 McFarland preparations at 24 h from the individual organisms was combined and further incubated in fresh TSB at 37 °C for 72 h. Notably, the culture medium of bacteria (TSB) was used in both KP and KP + CA to explore bacterial biofilms with and without fungi, as well as CA, as it can grow in the media for bacteria, as shown in our previous publications [25,26].

After that, the biofilms were analyzed using crystal violet staining (absorbance at 590 nm) and immunofluorescent staining. The fluorescent colors were used to detect microbial DNA (SYTO9) conjugated with Alexa Fluor (AF) 488 (Invitrogen, Waltham, MA, USA), the extracellular matrix (ECM) (Concanavalin) conjugated with AF647 (Invitrogen), and the fungal cell wall (calcofluor white) conjugated with AF460 (Sigma-Aldrich, St. Louis, MO, USA). Notably, DNA and ECM staining were used to determine the microbial abundance and biofilm protein matrix (both bacteria and fungi) [25,26], while fungal cell wall staining (binding to 1–3 β and 1–4 β polysaccharides of chitin) [69] represented the fungal abundance (chitin presented in the fungal cell wall but not in *Klebsiella* spp. [70]). Then, the visualization was performed using the LSM 800 Airyscan confocal laser scanning microscope (CLSM; Carl Zeiss, Jena, Germany) at a magnification of 1000× with z-stacking in 10 randomly selected fields, and fluorescent intensity was then analyzed using the ZEN imaging software (Carl Zeiss). For the biofilm organism burdens, the biofilms were dissolved and thoroughly vortexed in 1 mL of normal saline for 5 min before being directly incubated in TSA and DSA with chloramphenicol (Oxioid, Hampshire, UK) for 24 and 48 h, respectively, at 37 °C before colony enumeration.

### 4.2. RNA Extraction and Quantitative Real-Time Polymerase Chain Reaction

To estimate the biofilm-related genes of KP, biofilms were scraped and extracted using TRIzol reagent (Invitrogen, Waltam, MA, USA) according to the manufacturer’s instructions. Double-stranded cDNA was synthesized via one-step reverse transcription using the RevertAid First Strand cDNA kit (Thermo Fisher, Waltam, MA, USA). The biofilm-associated genes were evaluated by the QuantStudio 6 Flex Real-Time PCR (Applied Biosystem, Waltam, MA, USA) using the PowerUp SYBR Green Master Mix (Applied Biosystem, Waltam, MA, USA) following a previously published protocol [51,71,72]. Briefly, the housekeeping *16S rRNA* gene was used to normalize the transcriptional levels of the interesting genes with the comparative cycle threshold against the expression. The primers are listed in Table 1. The results were demonstrated using relative quantification with the comparative threshold method (ΔΔCt; 2^−ΔΔCt^), normalized by *β-actin* (an endogenous housekeeping gene).

### 4.3. Proteomic Analysis

Protein extraction and fractionation were prepared using a previously described method [25]. Briefly, the digested biofilms were pooled and fractionated using a high-pH reverse-phase peptide fractionation kit (Thermo Fisher Scientific, Waltham, MA, USA). Liquid chromatography–tandem mass spectrometry (LC-MS/MS) analysis of peptides was performed on an EASY-nLC1000 system coupled to a Q-Exactive Orbitrap Plus mass spectrometer equipped with a nano-electrospray ion source (Thermo Fisher Scientific, Waltham, MA, USA). Data acquisition and interpretation were monitored using Proteome DiscovererTM software 2.1 (Thermo Fisher Scientific, Waltham, MA, USA). Peptide spectra were analyzed using the SEQUEST-HT search engine against *K. pneumoniae* (strain ATCC13833) in the Swiss-Prot Database (509 proteins, March 2024). Biological process analysis of *K. pneumoniae* was carried out using DAVID Bioinformatics on 5 March 2024. Mass spectrometry proteomic data were determined and submitted to the ProteomeXchange consortium via PRIDE (http://www.proteomexchange.org) and are available under the project accession PXD050494.

### 4.4. In Vitro Experiments on Neutrophils

Male 8-week-old mice were intraperitoneally administered with 1 mL of 3% thioglycolate (Sigma-Aldrich) to activate neutrophils following previous publications [73]. Peritoneal neutrophils were collected after administration at 3 h and washed with ice-cold phosphate-buffered solution (PBS) before centrifugation at 1800 rpm with 4 °C for 5 min. The cells were resuspended in fresh RPMI 1640 medium (Invitrogen) supplemented with 10% heat-inactivated fetal bovine serum (FBS) (Invitrogen). The trypan blue exclusion method was used to determine the cell viability by counting unstained (live cells) versus stained cells (dead cells) using a hemocytometer. Only the samples with more than 95% cell viability were further used. Then, neutrophils (1 × 10^6^ cells/mL) were incubated with biofilms (KP, CA, and KP + CA) at an effector-to-target ratio (E:T) of 10:1 in 5% CO_2_ at 37 °C for 3 h. After that, trypan blue staining was used to determine neutrophil viability. The viable bacteria and fungi were determined using the dissolved biofilms in normal saline (1 mL) and thoroughly vortexed for 5 min and processed using the culture method for bacteria (TSA) and fungi (SDA) at 37 °C for 24 and 48 h, respectively, before colony enumeration. Supernatant cytokines (IL-1β, TNF-α, IL-6, and IL-10) and cell-free DNA were measured using an enzyme-linked immunosorbent assay (ELISA) (Invitrogen) and PicoGreen assay (Invitrogen), respectively, according to the manufacturer’s protocol [51,74]. The neutrophil extracellular traps (NETs) were also evaluated by measuring the expression of *peptidyl arginine deiminase 4* (*PAD4*), an important enzyme for NET formation [75], relative to *beta-actin* using PCR as described above, along with immunofluorescent staining with a previously published protocol [76]. Briefly, for NET detection by immunofluorescence, neutrophils were incubated with various antibodies, including (i) anti-neutrophil elastase (NE; ab68672) with goat anti-rabbit IgG-AF488 (ab150077) (green color), (ii) anti-myeloperoxidase (MPO; ab25989) with goat anti-mouse IgG-AF647 (ab150115) (red color), (iii) anti-citrullinated histone H3 (CitH3; ab5103) with donkey anti-rabbit IgG-AF647 (ab150075) (red color) (Abcam, Cambridge, MA, USA), and (iv) 4′,6-diamidino-2-phenylindole (DAPI) for nuclear staining (blue color). Visualization was performed by the LSM 800 (Carl Zeiss) and the fluorescent intensity was calculated using the ZEN imaging software (Carl Zeiss).

### 4.5. Animals and Animal Model

All animal experimental procedures were approved by the Institutional Animal Care and Use Committee (IACUC) of the Faculty of Medicine, Chulalongkorn University, Thailand (Approval Protocol No. 20/66), following the US National Institutes of Health (NIH) animal care and use protocol. Male C57BL/6 mice were purchased from Nomura Siam (Pathumwan, Bangkok, Thailand) and housed in clear plastic cages (3–5 mice per cage) in a standard mouse facility (12 h light/dark cycles, 25 °C temperature control, and 50 ± 10% relative humidity) using proper environmental enrichment (thick paper stripes) with free access to water and food (SmartHeart Rodent; Perfect Companion Pet Care, Bangkok, Thailand). For the pulmonary infection model [25], intraperitoneal injection of dexamethasone (0.1 mg/g of body weight) [77] was performed in 12-week-old mice for 5 consecutive days before intratracheal administration of 0.2 mL of *K. pneumoniae* (KP) or *C. albicans* (CA) at 1 × 10^8^ CFU/mL. For the combination of both organisms, 0.1 mL of the KP and CA preparations were combined, and normal saline administration was used as a control group. After intratracheal administration, mice were hourly observed for clinical symptoms for 3 h and at 6 and 12 h post-administration. The moribund mice were sacrificed according to the humane endpoint observation. All mice were euthanized at 24 h post-administration by cardiac puncture under isoflurane anesthesia before collecting samples, including blood, bronchoalveolar lavage fluid (BALF), and lung tissue. Serum creatinine and alanine transaminase were determined using a QuantiChrom creatinine assay (DICT-500) (BioAssay, Hayward, CA, USA) and EnzyChrom alanine transaminase assay (EALT-100) (BioAssay, Hayward, CA, USA), while cytokines and serum cell-free DNA were measured using ELISA (Invitrogen, Waltham, MA, USA) and the PicoGreen assay kit (Invitrogen, Waltham, MA, USA), respectively. Bacteremia and fungemia from blood and BALF were detected with the culture method using TSA and SDA for bacteria and fungi, respectively, before colony enumeration after incubation at 37 °C for 24 h. Neutrophil count (polymorphonuclear cells) in BALF was performed using a hematocytometer with trypan blue dye staining.

### 4.6. Histological Analysis and Immunofluorescent Imaging

The semi-quantitative evaluation of lung histology on paraffin-embedded samples was performed using hematoxylin and eosin (H&E) staining at 200× magnification in 10 randomly selected fields. The score of lung injury was estimated based on desquamation, dystelectasis/atelectasis, congestion, interstitial thickness, infiltration, and bronchial exudate with a modified lung injury score as follows: 0 (no injury), 1 (minimal/discrete), 2 (mild), 3 (moderate), and 4 (severe) [78]. In parallel, immunofluorescent histological analysis for NETs was prepared in the lung tissue kept in Tissue-Tek OCT compound (Sakura Finetek, Torrance, CA, USA) and incubated using all of the antibodies for NET detection as mentioned above. Then, the images were visualized by the LSM 800 (Carl Zeiss), and fluorescent intensity was calculated using the ZEN imaging software (Carl Zeiss).

### 4.7. Statistical Analysis

The mean ± standard error (SE) was used for data presentation, and differences between groups were examined for statistical significance using a one-way analysis of variance (ANOVA) followed by Tukey’s analysis or Student’s *t*-test for the comparison of multiple groups or two groups, respectively. Survival analysis was performed using the Log-rank test. All statistical analyses were performed with GraphPad software version 10.3 (La Jolla, San Diego, CA, USA). A *p*-value of < 0.05 was considered statistically significant.

## 5. Conclusions

Biofilms formed by the combination of *Klebsiella pneumoniae* (KP) and *Candida albicans* (CA) demonstrated greater biofilm biomass compared to those from KP alone, as indicated by crystal violet staining and biofilm thickness in fluorescent images. The biomass of KP + CA biofilms was mainly produced from CA, which demonstrated less prominent neutrophil activation and less severe pneumonia than KP alone, possibly due to some proteins as identified through proteomic analysis. The coexistence of bacteria and fungi can alter the natural history of disease; however, the complexity and heterogeneity among different pairs of specific bacteria and fungi, together with host immune responses, make it difficult to predict the outcomes in co-infection. More studies on this topic are required.

## Figures and Tables

**Table 1:** The primers used in the investigation.

Primer	Sequence	
16S rRNA	Forward	5′-TCCAGGTGTAGCGGTGAAAT-3′
	Reverse	5′-TGAGTTTTAACCTTGCGGCC-3′
mrkA	Forward	5′-CGATGCGAACGTTTACCTGT-3′
	Reverse	5′-TTCACGCCCAGTTTGCTTAC-3′
mrkD	Forward	5′-GCCAACATTAGCACCTCGTT-3′
	Reverse	5′-GTCGTCGGGCCATACTGATA-3′
wzi	Forward	5′-CAATGACCGGCTTCCTGATG-3′
	Reverse	5′- GCTGCTAAATGACTCAGGCC -3′
PAD4	Forward	5′-ACAGGTGAAAGCAGCCAGC-3′
	Reverse	5′-AGTGATGTAGATCAGGGCTTGG-3′
β-actin	Forward	5′-CGGTTCCGATGCCCTGAGGCTCTT-3′
	Reverse	5′-CGTCACACTTCATGATGGAATTGA-3′

## Data Availability

No new data were created or analyzed in this study. Data sharing is not applicable to this article.
